# RETRATAÇÃO PARCIAL

**DOI:** 10.36660/abc.20220705.RETRAT

**Published:** 2023-12-19

A equipe editorial da revista Arquivos Brasileiros de Cardiologia comunica a publicação formal de Retratação Parcial do artigo:

Lypovetska, Sofiya. Fenótipos MINOCA Um Desafio para o Manejo Específico do Paciente. Arq Bras Cardiol. 2023; 120(6):e20220705.

https://doi.org/10.36660/abc.20220705

Desde que foi comprovado plágio da figura.

Prof. Dr. Carlos Eduardo Rochitte

Editor-chefe

