# Supplementary material for: RETRATAÇÃO PARCIAL
Source: Arq Bras Cardiol. 2023 Dec 19;120(12):e20220705. [Article in Portuguese] doi: 10.36660/abc.20220705.RETRAT (PMC11107420; doi:10.36660/abc.20220705.RETRAT)
Supplement: Translation

# Translation: PARTIAL-RETRACTION

The editorial team of the Arquivos Brasileiros de Cardiologia journal hereby announces the formal publication of a Partial-Retraction for the article:

Lypovetska, Sofiya. MINOCA Phenotypes - A Challenge for Patient-Specific Management. Arq Bras Cardiol. 2023; 120(6):e20220705.

https://doi.org/10.36660/abc.20220705

As plagiarism of the figure has been confirmed.

Prof. Dr. Carlos Eduardo Rochitte

Editor-in-chief
